# Chlorinated Water Modulates the Development of Colorectal Tumors with Chromosomal Instability and Gut Microbiota in *Apc*-Deficient Mice

**DOI:** 10.1371/journal.pone.0132435

**Published:** 2015-07-17

**Authors:** Tatsunari Sasada, Takao Hinoi, Yasufumi Saito, Tomohiro Adachi, Yuji Takakura, Yasuo Kawaguchi, Yusuke Sotomaru, Kazuhiro Sentani, Naohide Oue, Wataru Yasui, Hideki Ohdan

**Affiliations:** 1 Department of Gastroenterological and Transplant Surgery, Applied Life Science, Institute of Biomedical & Health Sciences, Hiroshima University, Hiroshima, Japan; 2 Natural Science Center for Basic Research and Development, Hiroshima University, Hiroshima, Japan; 3 Department of Molecular Pathology, Hiroshima University Institute of Biomedical and Health Sciences, Hiroshima, Japan; Colorado State University, UNITED STATES

## Abstract

The gastrointestinal tract is continuously exposed to a variety of chemicals and commensal bacteria. Recent studies have shown that changes in gut microbial populations caused by chlorine or other chemicals in the drinking water influence the development of human colorectal cancer, although the mechanism of tumorigenesis in the gut epithelium is obfuscated by the diversity of microflora and complexity of the tumor microenvironment. In this regard, mouse models that recapitulate human colorectal cancer are an invaluable tool. In this study, we used two conditional adenomatous polyposis coli (*Apc*) knockout mouse models to investigate the effect of chlorinated water on tumorigenesis in the digestive tract. Mice with colon-specific carcinoma—caused by either chromosomal (*CDX2P 9.5-NLS Cre;Apc^+/flox^*, abbreviated to *CPC;Apc*) or microsatellite (*CDX2P9.5-G19Cre;Apc^flox/flox^ and CDX2P9.5-G22Cre;Apc^flox/flox^*) instability, respectively—were administered chlorinated (10.0 mg/L chlorine) or tap (0.7 mg/L chlorine) water and evaluated for colon polyp formation. In *CPC;Apc* mice given chlorinated drinking water, tumors tended to develop in the colon, whereas in those that drank tap water, tumors were mostly observed in the small intestine. There was no difference in the rate of tumor formation of *CDX2P9.5-G19Cre;Apc^flox/flox^ and CDX2P9.5-G22Cre;Apc^flox/flox^* mice consuming chlorinated as compared to tap water, suggesting that microsatellite instability in the *Apc* gene does not significantly affect tumorigenesis. Chlorinated water altered the enteric environment by reducing the fecal populations of the obligatory anaerobes *Clostridium perfringens* and *C*. *difficile*, as well as species belonging to the *Atopobium *cluster, including *Enterobacteriaceae* and *Staphylococcus* sp., which was associated with colon tumorigenesis in *CPC;Apc *mice. These results suggest that differences in tumorigenesis among *CPC;Apc* mice consuming chlorinated versus tap water may be due to differences in gastrointestinal commensal populations.

## Introduction

Intestinal microflora comprises ~10^15^ bacteria, representing more than 400 bacterial species. Intestinal microflora modulates metabolic functions of nutrient absorption, trophic effects on the intestinal epithelium, and protection against alien microbes, and it is thus closely related to human health and disease [1]. The classic example of control of bacterial distribution in the gastrointestinal tract is the gastric acid barrier and progression of *Helicobacter pylori*-induced chronic atrophic gastritis [2]. Similarly, the bacterial distribution in the colon epithelium influences chronic enteritis and colorectal carcinomas, although colon cancer has not been linked to specific intestinal bacteria. The possible association between substances in drinking water and increasing rates of human cancer has been explored [3], and the addition of chlorine to drinking water has been found to alter the enteric environment and mediate the development of colon cancer.

The acquisition of genomic instability is a crucial feature in the development of human cancer. Chromosomal instability (CIN) and microsatellite instability (MSI) are two distinct pathways in colorectal cancer. A current theory is that human colon cancers arise from adenomatous precursors, which is observed in benign adenomas and increases in tandem with tumor progression by the accumulation of gain-of-function mutations in proto-oncogenes and loss-of-function mutations in tumor suppressor genes. The identification of heterozygous germline mutations in familial adenomatous polyposis (FAP) syndrome has highlighted the role of the adenomatous polyposis coli (*APC*) gene in sporadic tumor development and has clarified mechanisms by which colon tumors arise [4–6].

The C57BL6/J mouse model of multiple intestinal neoplasia [5, 7] carrying the *Apc* ^*Min*^ germline mutations develops ~50 adenomas and a few carcinomas in the small intestine by the age of 120–140 days [8]. *Apc* ^*Min*^ mice are commonly used to study intestinal tumorigenesis and to test cancer prevention and treatment strategies [9]; however, *Apc* ^*Min*^ mice have notable disadvantages as models for human colorectal carcinogenesis. For instance, they develop tumors mainly in the small intestine, while the majority of human gastrointestinal tumors are found in the colon and rectum. *Apc* ^*Min*^ mice usually die of intestinal obstruction or anemia by the age of 140 days, and few adenomas develop into carcinomas. To overcome these limitations, we developed two distinct mouse models of spontaneous carcinoma with colon-preferential *Apc* inactivation.

*C* *DX2* *P* *9*.*5-NLS* *C* *re;Apc* ^*+/flox*^ (*CPC;Apc*) mice develop adenomas and carcinomas mainly in the distal colon and rectum, with a small number of cecum and small intestine adenomas [10]. In the human colorectal carcinoma with the CIN phenotype, loss of heterozygosity (LOH) at loci on chromosomes 5q, 17p, and/or 18q is frequent, while *CPC;Apc* mice carrying constitutional, heterozygous, inactivating mutations in the *Apc* gene have shown that the wild-type *Apc* allele is inactivated by LOH, demonstrating that CIN contributes to tumor progression.

*CDX2P9*.*5-G19Cre;Apc* ^*flox/flox*^ and *CDX2P9*.*5-G22Cre;Apc* ^*flox/flox*^ mice carry a bi-allelic *Apc* inactivation mutation in the colon epithelium, resulting from a sporadic activation of Cre recombinase transgenes with either 19 or 22 guanine nucleotides (G19Cre or G22Cre, respectively) introduced downstream of the initiating ATG codon, followed by a frameshift reversion mutation in mononucleotide repeats [11]. These mice develop large numbers of polypoid lesions in the proximal colon, thus demonstrating adenomatous changes [11]. Microsatellites are short tandem repeats that are widely distributed throughout eukaryotic genomes. Replication of these repeats is error-prone because of nucleotide slippage during synthesis, creating insertion/deletion loops [12]. The loss of nucleotide mismatch repair results in the accumulation of microsatellite repeats of variable lengths, and thus, MSI might be responsible for tumorigenesis in *CDX2P9*.*5-G19Cre;Apc* ^*flox/flox*^ and *CDX2P9*.*5-G22Cre;Apc* ^*flox/flox*^ mice.

We sought to develop an approach to determine how the chlorine in drinking water affects tumorigenesis in two mouse models with distinct genomic instability pathways. We also attempted to identify the underlying mechanism and gut microbiota responsible for the regulation of tumor development.

## Materials and Methods

### Mice

Breeding colonies were established using C57BL/6J mice (CLEA Japan, Tokyo, Japan). Embryos of *C* *DX2* *P* *9*.*5-NLS* *C* *re (CPC)* mice, *Apc* ^*flox/flox*^ mice [13], and *R26R* reporter mice [14] were obtained from the University of Michigan. These embryos were transferred to pseudopregnant C57BL/6J mice. Eight-week-old *Apc* ^*flox/flox*^ females were bred with male *CDX2P 9*.*5-NLS Cre* males. To avoid sex bias, only male *CPC;Apc* mice were used in this study. *CDX2P9*.*5-G19Cre* and *CDX2P9*.*5-G22Cre*[11] with C57BL6/J background were obtained from plasmids. All mice were housed under specific pathogen-free conditions. Mice were fed Teklad Mouse Breeder Diet 8626. The breeding room was maintained at a constant temperature of 23°C ± 2°C, relative humidity of 50% ± 5%, 15–20 air changes per hour, and a 12-h light/dark cycle, with lights on at 8:00 am. Four or five mice were housed per cage with chopped wood bedding. All animal protocols were approved by the Institutional Animal Care and Use Committee at our institution (Hiroshima University). Mice were euthanized by CO_2_ asphyxiation as per IACUC guidelines.

Loss of *Apc* heterozygosity was assessed by multiplex PCR with the following primers: Apc-P3, 5′-GTTCTGTATCATGGAAAGATAGGTGGTC-3′; Apc-P4, 5′-CACTCAAAACGCTTTTGAGGGTTGATTC-3′; and Apc-P5, 5′-GAGTACGGGGTCTCTGTCTCAGTGAA-3′. The target (580S), deletion (580D), and wild-type alleles yielded products of 314 bp (P3 and P4), 258 bp (P3 and P5), and 226 bp (P3 and P4), respectively [13]. The presence of the CDX2 promoter region was assessed by PCR with the following primers: CDX2P-10B-S, 5′-CCGACCTTTACATGTGAGCG-3ʹ; and CDX2P-10B-AS, 5ʹ-CACTGCAATCTCGCTTCATTC-3ʹ.

### Animal treatment and tissue harvesting

Male *CPC;Apc* mice (n = 9–10 per group) and male *CDX2P9*.*5-G19Cre;Apc* ^*flox/flox*^ and *CDX2P9*.*5-G22Cre;Apc* ^*flox/flox*^ mice (n = 3 per group) received the Teklad Mouse Breeder Diet 8626 from week 3 to the end of their life. Tap water (0.7 mg/L chlorinated water) was provided to the control group, and 10.0 mg/L chlorinated water was provided to the study group. *CPC;Apc* mouse were sacrificed at 40 weeks of age, and *CDX2P9*.*5-G19Cre;Apc* ^*flox/flox*^ and *CDX2P9*.*5-G22Cre;Apc* ^*flox/flox*^ mouse were at 15 weeks of age by cervical dislocation under anesthesia. The entire gastrointestinal tract was removed immediately after sacrifice and flushed with phosphate-buffered saline (PBS). Intestinal tissue was sliced longitudinally, and the location; multiplicity; and diameters of polyps in the small intestine, cecum, and colon were recorded. The intestine was transferred to 10% buffered formalin to be processed for histopathological studies. Consistent with the histologic appearance, a hemispherical shape was assumed for small and large bowel polyps (volume = 2/3πr^3^, r = radius).

### Fecal bacteriologic examinations

Fecal samples for bacteriological analysis were acquired from pre-treated mice. Immediately after defecation, fecal samples were weighed and then suspended in 9 volumes of RNAlater (Ambion Inc., Austin, TX, USA), an RNA stabilization solution. Next, the preparations were incubated for 10 min at room temperature. For RNA stabilization, fecal homogenate (200 μL) was added to 1 mL sterilized PBS and centrifuged at 5,000 × *g* for 10 min. The supernatant was discarded and the pellet stored at −80°C until RNA extraction. RNA was isolated using a modified method of acid guanidinium thiocyanate-phenol-chloroform extraction. Finally, the nucleic acid fraction was suspended in 1 mL nuclease-free water (Ambion)[15, 16]. To determine bacterial number by reverse transcription-quantitative polymerase chain reaction (RT-qPCR), a standard curve was generated from RT-qPCR data (using the threshold cycle [C_T_], i.e., the cycle number when threshold fluorescence was reached) and the corresponding cell count, which was determined microscopically with 4,6-diamidino-2-phenylindole (DAPI) (Vector Laboratories, Burlingame, CA) staining [17] for the dilution series of the standard strains [15, 16]. To measure the bacterial populations in each sample, 3 serial dilutions of extracted RNA were used for RT-qPCR. C_T_ values in the linear range of the assay were applied to the standard curve (generated in the same experiment) to obtain the corresponding bacterial cell count in each nucleic acid sample and then converted to the number of bacteria per sample. The specificity of the RT-qPCR assay using group- or species-specific primers was determined as described in earlier studies [15, 16].

### Tissue fixation, staining, and galactosidase quantitation

Dissected gastrointestinal tissues were opened and washed with PBS containing 0.01% Triton X-100 at 4°C with agitation. After brief fixation with 4% paraformaldehyde containing 1.25 mmol/L EGTA and 2 mmol/L MgCl_2_ in PBS, tissues were placed in 5-bromo-4-chloro-3-indolyl-d-galactopyranoside (X-gal) staining solution [1 mg/mL X-gal in N,N dimethylformamide, 5 mmol/L K_3_Fe(CN)_6_, 5 mmol/L K_4_Fe(CN)_6_, 2 mmol/L MgCl_2_, 1.25 mmol/L MgCl_2_ in PBS] for 4–12 h at 37°C. The tissue was fixed with 0.2% glutaraldehyde for 10 min and with 4% paraformaldehyde for 4 h at 4°C. Tissues were then stored in 70% ethanol. For quantitative analysis of X-gal expression, grayscale 2-gradation 45× stereoscopic microimages were evaluated.

### Microdissection

Formalin-fixed, paraffin-embedded tissues were sectioned (10 μm), mounted on MembraneSlides (Leica Microsystems, Wetzlar, Germany), and then weakly stained with hematoxylin and eosin. Specific regions (neoplastic versus non-neoplastic tissue) were dissected by laser microdissection with a Leica AS LMD (Leica Microsystems). Microdissected tissues were collected at the bottom of the tube and incubated overnight in digestion buffer (10 mM Tris-HCl pH 8.0, 1% Tween-20) with 200 μg proteinase K at 55°C to extract DNA.

### MSI analysis

MSI was assessed in tumors from *CPC;Apc* and *CDX2P9*.*5-G19Cre;Apc* ^*flox/flox*^ mice with 4 previously described mononucleotide repeats (*mBat-26*, *mBat-37*, *D7Mit91*[18], *GA29*[19]). Primers for PCR amplification were as follows: *mBat26*-S, 5′-TCACCATCCATTGCACAGTT-3′; *mBat-26*-AS, 5′-CTGCGAGAAGGTACTCACCC-3′; *mBat-37*-S, 5′-TCTGCCCAAACGTGCTTAAT-3′; *mBat-37-*AS, 5′-CCTGCCTGGGCTAAAATAGA-3′; *D7Mit91*-S, 5′-TCTTGCTTGCATACACTCACG-3′; *D7Mit91*-AS, 5′-GAGACAAACCGCAGTCTCCT-3′; *GA29*-S, 5′-CAGGAGGTCAAGGTCATCCTAAG-3′; and *GA29*-AS, 5′-CCACCATGGTAGGAGCTTGCTA-3′. The forward primers were synthesized by Applied Biosystems and labeled with 6-FAM (*mBat26*), VIC (*mBat-37*), PET (*D7Mit91*), and NED (*GA29*). Amplification of mononucleotide repeats was performed with fluorescence-labeled primers in 25-μL reactions containing 12.5 μL Quick Taq HS Dye (Toyobo, Tokyo, Japan), 10 μM primers, and 1–2 ng DNA. PCR was performed on a PE 9600 Thermal Cycler (Applied Biosystems, Foster City, CA) with the following cycling profile: 1 cycle of 94°C for 5 min; 40 cycles of 94°C for 30 s, 55°C for 30 s, 72°C for 30 s; 1 cycle of 72°C for 10 min; and a final hold at 4°C. Separation and detection of amplified fragments were performed on an ABI PRISM1 3100 Genetic Analyzer according to manufacturer protocols (Applied Biosystems, Foster City, CA). The size marker was the GeneScan 600 LIZ Size Standard.

## Results

### Chlorinated drinking water increases colon polyp formation in *CPC;Apc* mice with CIN

**Fig 1:**
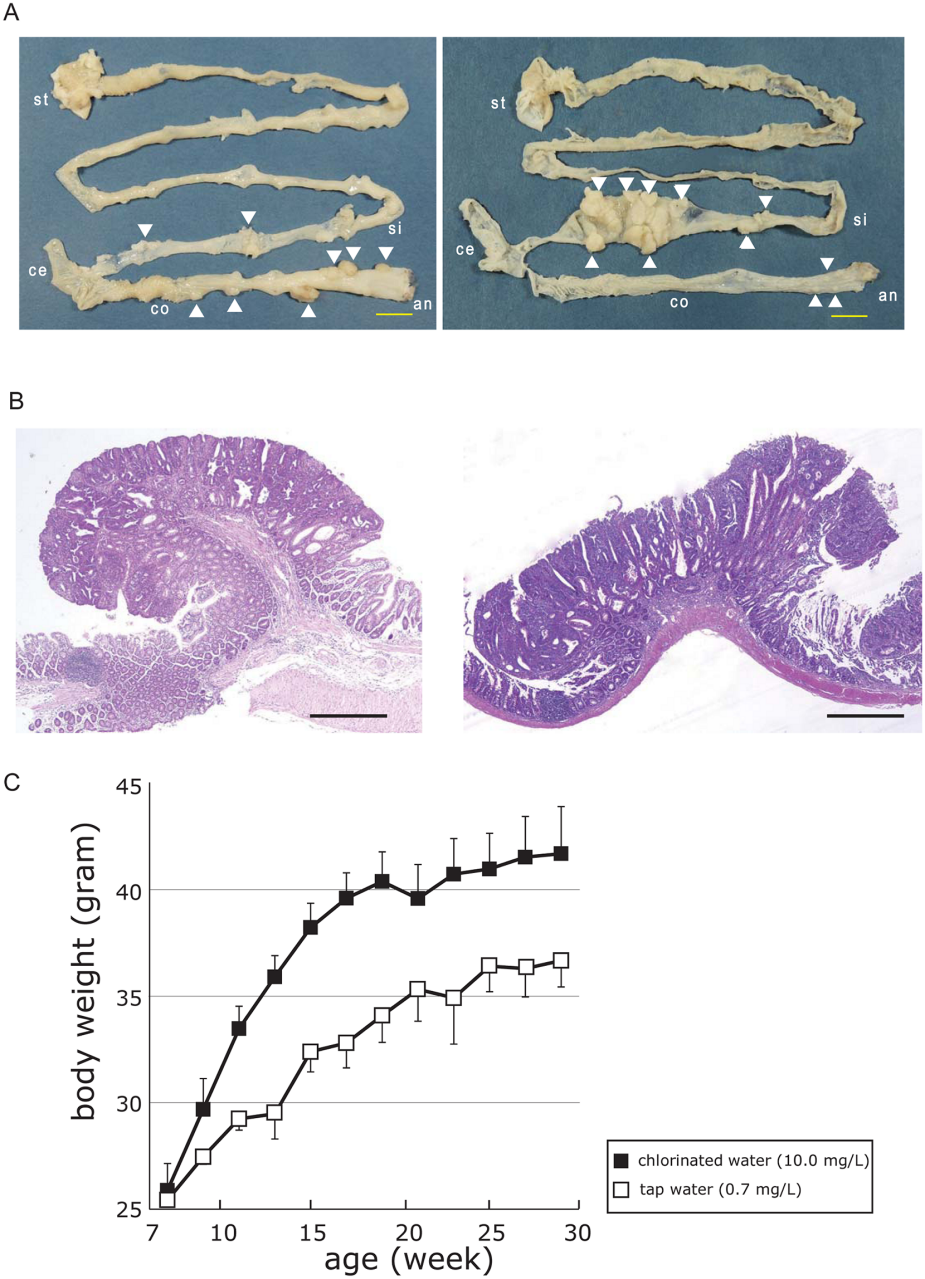
Tumorigenesis and body weight in *CPC;Apc* mice. **A,** Tumorigenesis in 40-week-old *CPC;Apc* mice. Left, mouse administered chlorinated water (10.0 mg/L chlorine). Right, mouse administered tap water (0.7 mg/L chlorine). Tumors were mainly detected in the colon and at the end of the ileum in the chlorinated and tap water groups, respectively. st, stomach; je, jejunum; ce, cecum; co, colon; an, anus. Bar, 10 mm. **B,** Tumor histology in *CPC;Apc* mice. H&E staining of a colonic tumor from a chlorinated water-treated mouse (left) and a small intestinal tumor from a tap water-treated mouse (right). Bar, 500 μm. **C,** Body weight gain in the chlorinated water group (black box) was the same as in wild-type C57B/6 mice, while the tap water group (white box) had significantly lower weight gain. *P < 0.05 (ANOVA).

**Fig 2:**
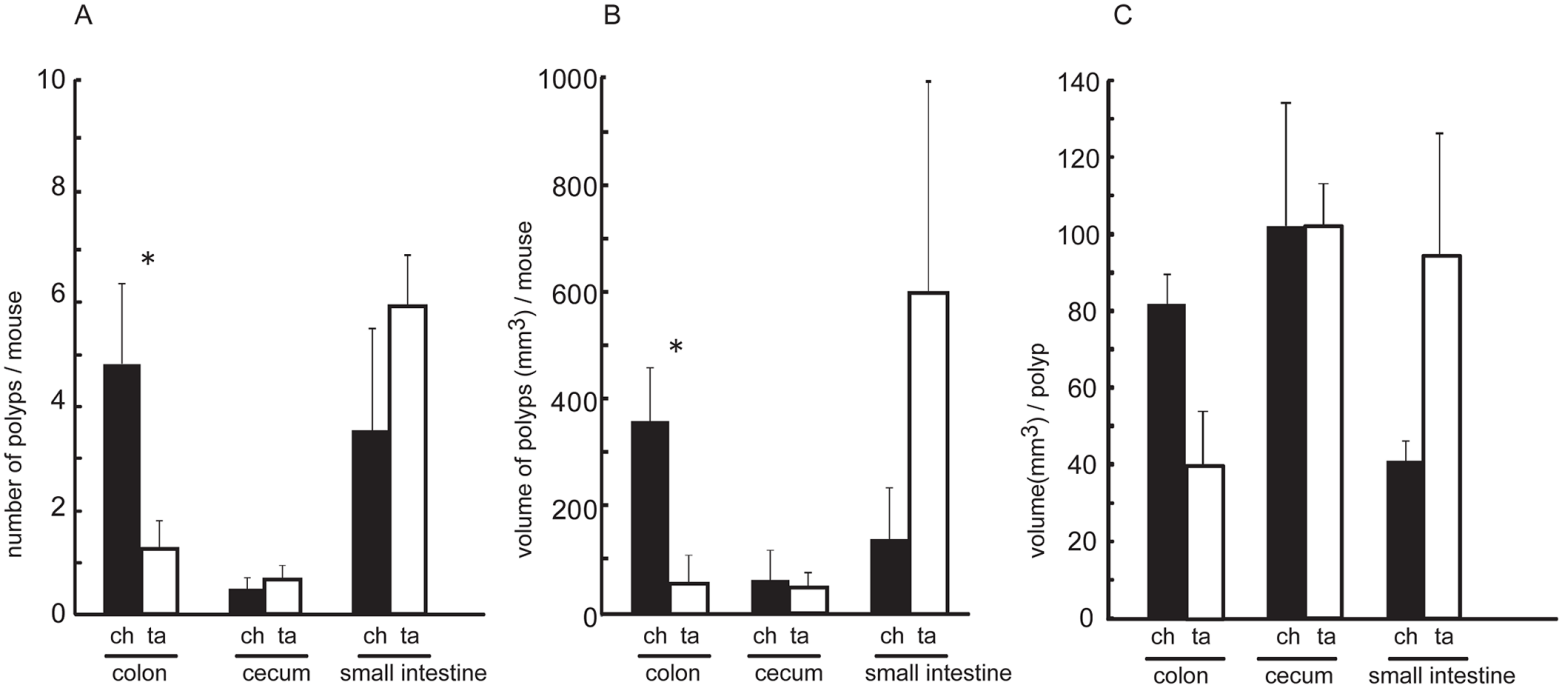
Polyp number and volume in the colon, cecum, and small intestine. A greater number of polyps was detected in the colon than in the ileum of the chlorinated water group (black columns), whereas more polyps occurred in the small intestine in the tap water group (white columns). Polyp volume was correlated with polyp number, and colon polyp volume differed significantly between the two groups. ch; chlorinated water group, ta; tap water group. *P < 0.05 (Student’s t test).

We investigated the effect of chlorinated water on tumorigenesis in the colon and small intestine in a mouse model of intestinal tumor formation by CIN. *CPC;Apc* mice harbor an *Apc* mutation that is predominantly manifested in the colon epithelium [10]. We compared the appearance of colon, cecum, and small intestine polyps in chlorinated water- and tap water-treated mice sacrificed at 40 weeks of age by examining the entire intestinal tract in each mouse. The consumption of chlorinated and tap water was associated with increased tumor multiplicity in the colon and distal small intestine, respectively (Fig 1A). Small intestinal tumors in the tap water group were large, polypoid, and non-invasive. In contrast, the chlorinated water group exhibited raised, peduncular colonic tumors in which relatively well-differentiated, epithelia-rich lesions with tubular glands were attached to the mucosa by stroma-rich stalks (Fig 1B). The increase in body weight was greater in chlorinated water-treated than in tap water-treated *CPC;Apc* mice (Fig 1C). Quantitative analysis revealed the presence of a greater number of colonic polyps (~4-fold higher) in mice treated with chlorinated as compared to tap water; small intestinal polyps were more numerous in the latter (Fig 2A), although the difference between the groups was not significant. Colon tumor volumes per mouse were also greater in the chlorinated water than in the tap water group. There were no between-group differences for cecal and small intestinal tumors (Fig 2B), and polyp volumes did not differ between the colon, cecum, and small intestine (Fig 2C). Thus, polyps occurred mostly in the colon in mice that consumed chlorinated water, and similar numbers of polyps were present in the cecum and small intestine of the two groups. In the tap water group, congested tumors with high volume in the distal small intestine obstructed the small bowel, likely accounting for the more modest gain in body weight (Figs 1C and 2B).

### Tumors from *CDX2P9*.*5-G19Cre;Apc* ^*flox/flox*^ mice have a hypermutable phenotype

**Fig 3:**
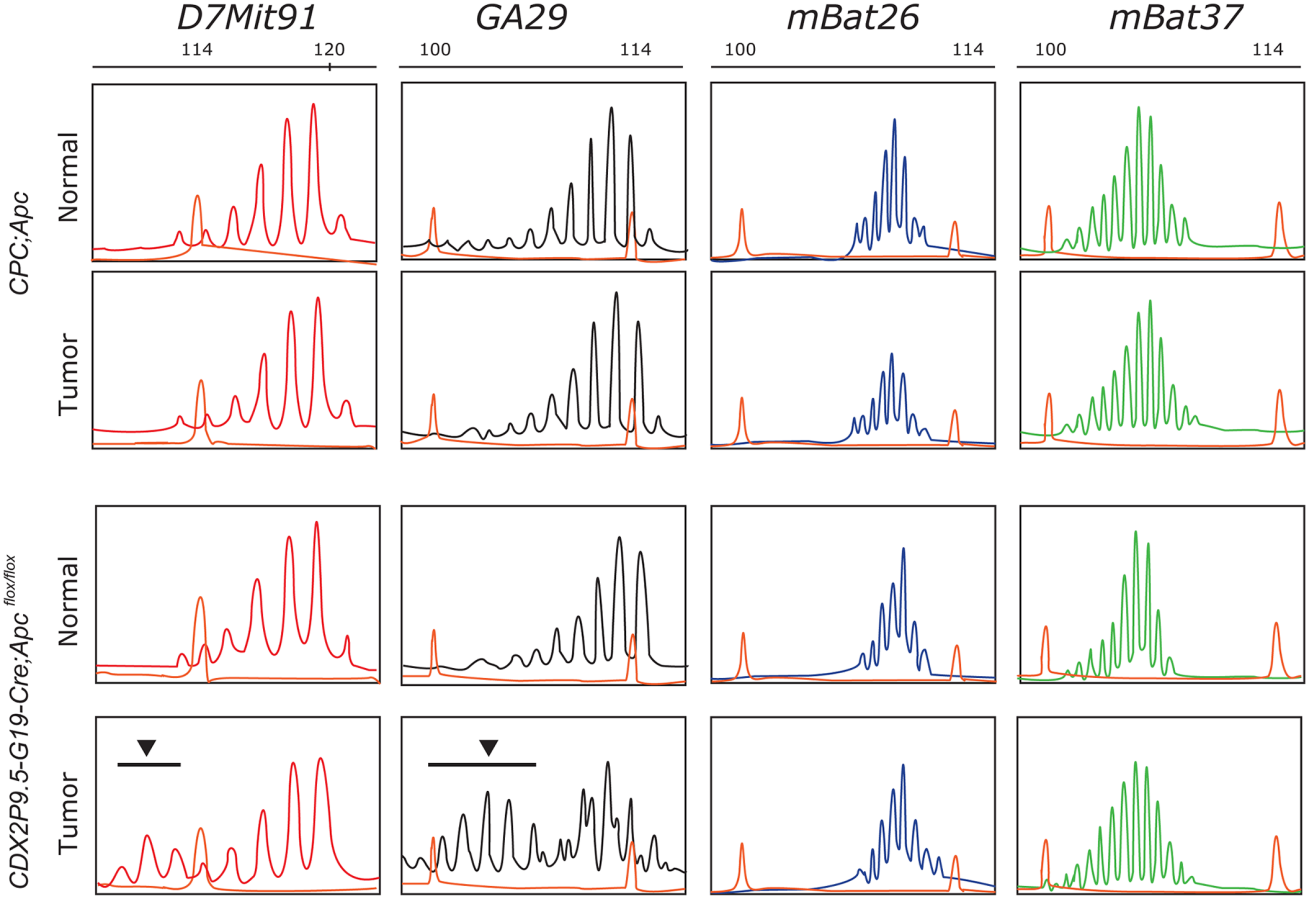
MSI in tumors from *CPC;Apc* and *CDX2P9*.*5-G19Cre;Apc* ^*flox/flox*^ mice. Tumors from *CDX2P9*.*5-G19Cre;Apc* ^*flox/flox*^ mice showed a hypermutable phenotype (arrowhead and black bar) for two (*D7Mit91* and *GA29)* of four markers investigated, suggesting that tumor development occurs via MSI. The 600 LIZ size standard (Eurofins GeneScan, Frieburg, Germany) was used as a marker for sizing DNA fragments.

We investigated the effect of chlorinated water on tumorigenesis associated with MSI, another form of genomic instability in colorectal cancer. We speculated that mono- or dinucleotide repeats would be more susceptible to MSI in tumors from *CDX2P9*.*5-G19 Cre;Apc* ^*flox/flox*^ mice than in those from *CPC;Apc* mice with CIN. The MSI status of tumors from *CDX2P9*.*5-G19Cre;Apc* ^*flox/flox*^ mice was determined from two representative mononucleotide (*mBat26* and *mBat37*) and dinucleotide *(D7Mit91* and *GA29)* repeat markers by short tandem repeat scanning (Fig 3). Mutations were detected in two (*D7Mit91* and *GA29*) of the four markers in tumors from *CDX2P9*.*5-G19Cre;Apc* ^*flox/flox*^ mice (Fig 3), but there were no mutations in the four markers in tumors from *CPC;Apc* mice, suggesting that tumors in *CDX2P9*.*5-G19Cre;Apc* ^*flox/flox*^ mice have a hypermutable phenotype that is presumably caused by the loss of DNA mismatch repair activity, which initiates sporadic activation of Cre recombinase by a frameshift reversion mutation.

### Chlorinated water has no effect on the initiation or development of tumors with MSI

**Fig 4:**
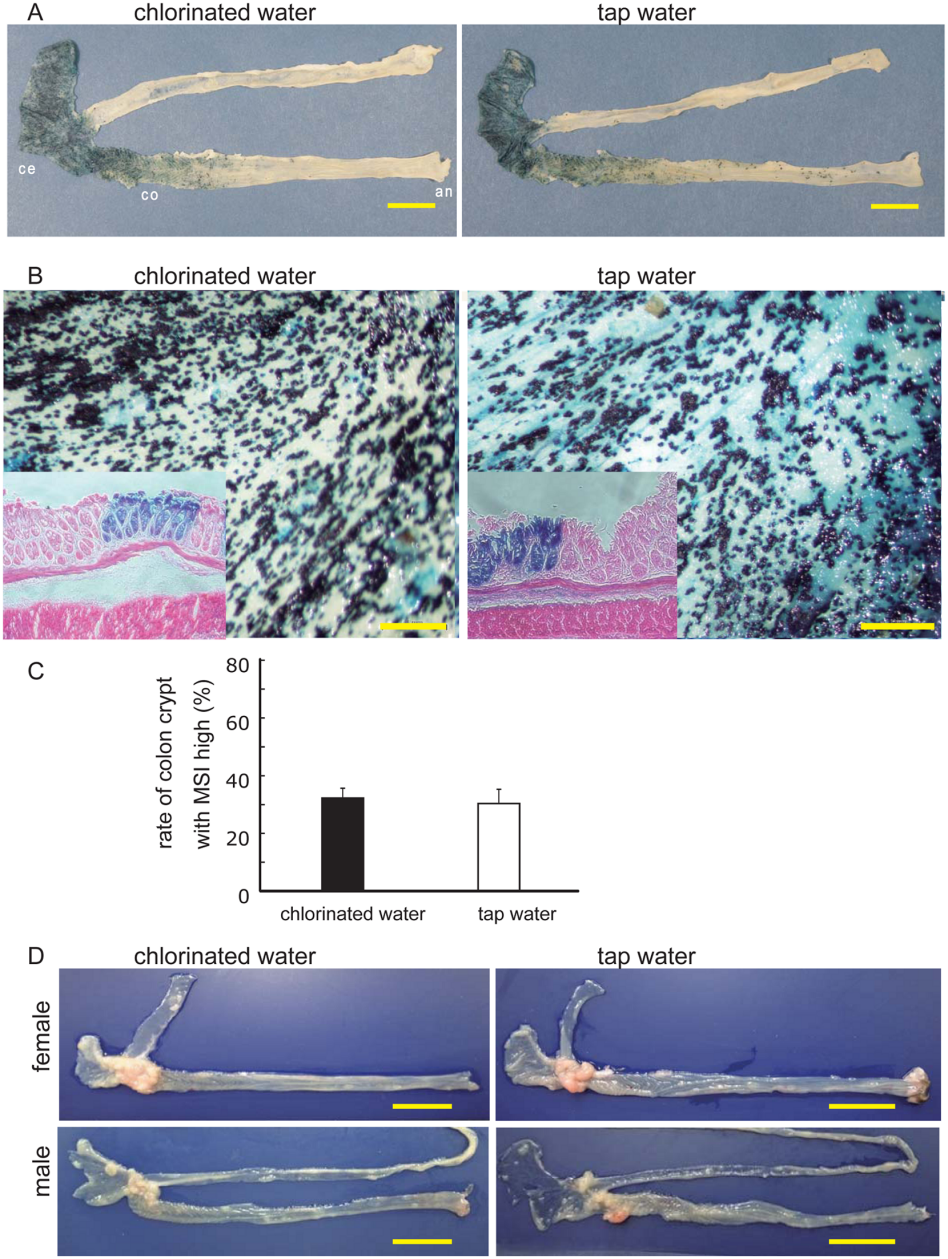
Cre activation in *CDX2P9*.*5-G19Cre;R26R* mice. **A,** X-gal activity in cecal crypt in a 20-week-old *CDX2P9*.*5-G19Cre;R26R* mouse. ce, cecum; co, colon; an, anus. Bar, 10 mm. **B,** Stereomicrograph (45×) of the cecal epithelium (small box) with X-gal and eosin staining of a 20-week-old *CDX2P9*.*5-G19Cre;R26R* mouse. Bar, 1 mm. **C,** Quantitative analysis of X-gal staining in 20-week-old *CDX2P9*.*5-G19Cre;R26R* mice. Cells were counted in six fields; data represent mean ± SD. **D,** Dissected ileum and colorectal tract of *CDX2P9*.*5-G22 Cre;Apc* ^*flox/flox*^ mice with cecum-proximal tumors. Left, tissue sample from a 15-week-old mouse treated with chlorinated water (10.0 mg/mL). Right, tissue sample from a 15-week-old mouse treated with tap water (0.7 mg/mL). Bar, 10 mm.

To further investigate the influence of chlorinated water on MSI status in the gastrointestinal epithelium, we used *Rosa26R* mice as the Cre reporter strain [14]. *CDX2P9*.*5-G19Cre* and *CDX2P9*.*5-G22Cre* mice were crossed with *R26R* mice to generate *CDX2P9*.*5-G19 Cre;R26R* and *CDX2P9*.*5-G22Cre;R26R* lines [11], respectively. These were used to monitor the rate of Cre-mediated recombination, which causes a frameshift reversion mutation by MSI in a subset of cells, suggesting a hypermutable phenotype that arose from a single-nucleotide (G19) microsatellite sequence in the colon epithelium. *CDX2P9*.*5-G19 Cre; R26R* mice were sacrificed at 20 weeks of age and X-gal staining was carried out on gastrointestinal tract tissue; the small intestine and colon of these mice are shown in Fig 4A. In the cecal epithelium, X-gal staining was observed only in the epithelial layer (Fig 4B), but there was no difference in terms of X-gal staining intensity relative to staining in the colon crypt between chlorinated and tap water-treated groups of this strain (Fig 4C) and in *CDX2P9*.*5-G22Cre;R26R* mice (data not shown).

To confirm the effect of chlorinated water on tumorigenesis in the MSI-associated hypermutable phenotype, *CDX2P9*.*5-G19Cre;Apc* ^*flox/flox*^ and *CDX2P9*.*5-G22Cre;Apc* ^*flox/flox*^ mice were administered chlorinated or tap water for 20 weeks. There was no difference in colon tumor development between the two strains (Fig 4D), consistent with previous data from R26R mice. These results strongly suggest that chlorinated water does not influence hypermutability and tumorigenesis due to MSI in the mouse colonic epithelium.

### *C*. *perfringens* abundance is reduced by chlorinated water consumption

To investigate the effect of chlorinated water on gut microbiota, feces were obtained directly from the colon of four *CPC;Apc* mice in each treatment group. A bacteriological analysis demonstrated that the number of *C*. *perfringens* (an obligate anaerobe) was significantly lower (P = 0.002) in mice that drank chlorinated as opposed to tap water (Table 1). Among species in the *Atopobium* cluster, *C*. *difficile*, *Enterobacteriaceae*, and *Staphylococcus* counts were reduced in the chlorinated water group and *C*. *perfringens* was undetectable, in contrast to the tap water group. Thus, the elimination of *C*. *perfringens* was found to be associated with colon tumorigenesis, suggesting an anti-tumorigenic role for *C*. *perfringens*. Taken together, our findings suggest that the reduced sizes of specific anaerobe populations caused by the consumption of chlorinated water could promote tumor development in the colon via a mechanism involving CIN as opposed to MSI.

**Table 1:** Fecal bacteria in mice treated with chlorinated or tap water*.

	Chlorinated water				Tap water				P value
**Total bacteria**	10.8	10.9	10.7	9.7	10.6	10.9	11.7	10.9	0.219
**Obligatory anaerobe**									
* Clostridium coccoides* group	10.6	10.5	10.3	9.3	9.8	10.7	10.8	9.8	0.814
* C*. *leptum* subgroup	9.7	10.2	9.6	9.0	9.7	9.7	10.5	9.5	0.522
* Bacteroides fragilis* group	9.9	10.3	10.0	8.3	10.0	9.9	11.0	10.2	0.253
* Bifidobacterium*	10.0	8.2	10.1	8.2	10.0	9.3	11.3	10.7	0.132
* Atopobium* cluster	8.7	8.4	8.8	7.8	9.3	8.9	10.2	9.5	0.025
* Prevotella*	8.8	9.4	8.6	6.9	7.7	8.7	10.0	8.2	0.768
* C*. *difficile*	5.6	5.3	6.0	4.9	6.4	5.9	6.6	5.9	0.043
* C*. *perfringens*	< 2.0	< 2.0	< 2.0	< 2.0	7.9	4.5	9.4	7.7	0.002
**Facultative anaerobe**									
Total *Lactobacillus*	9.9	8.4	9.5	9.0	8.6	9.9	10.1	8.4	0.930
* L*. *acidophilus* subgroup	9.6	6.1	9.1	8.8	6.0	9.8	8.0	7.5	0.623
* L*. *brevis*	4.2	4.5	4.7	< 2.7	4.0	4.2	5.5	4.6	0.366
* L*. *casei* subgroup	< 4.0	< 4.0	< 4.0	< 4.0	< 4.0	< 4.0	< 4.0	< 4.0	-
* L*. *fermentum*	< 4.2	< 4.2	< 4.2	< 4.2	< 4.2	< 4.2	< 4.2	< 4.2	-
* L*. *fructivorans*	< 3.0	< 3.0	< 3.0	< 3.0	< 3.0	< 3.0	< 3.0	< 3.0	-
* L*. *plantarum* subgroup	< 2.0	5.0	4.1	< 2.0	< 2.0	< 2.0	< 2.0	< 2.0	0.144
* L*. *reuteri* subgroup	9.6	6.2	8.4	8.5	5.7	9.3	7.6	6.9	0.469
* L*. *ruminis* subgroup	9.0	8.4	9.1	7.7	8.6	8.5	10.1	8.3	0.558
* L*. *sakei* subgroup	4.6	< 2.2	4.3	5.3	2.5	3.9	5.0	4.0	0.777
* Enterobacteriaceae*	5.1	< 4.5	5.8	<4.5	6.8	6.2	8.5	7.2	0.009
* Enterococcus*	8.6	8.4	8.6	8.2	8.4	8.1	9.6	8.0	0.850
* Staphylococcus*	8.2	8.9	8.2	7.0	6.5	6.4	7.4	6.8	0.029
**Aerobes**									
*Pseudomonas*	< 3.0	< 3.0	< 3.0	< 3.0	< 3.0	< 3.0	< 3.0	< 3.0	-

*Mean bacterial counts (log_10_ cells/g) per 1 g of feces from four mice in each group.

## Discussion

The current study demonstrated the effect of long-term intake of chlorinated drinking water on spontaneous intestinal and colonic tumorigenesis in two different conditional *Apc* knockout mouse models that recapitulate distinct genomic instability phenotypes, namely CIN and MSI. Epidemiological cohort studies have found an association between chlorinated drinking water and increased incidence of colorectal cancer [20, 21], while animal studies have suggested that high concentrations of certain chemicals in chlorinated drinking water increase the incidence of colorectal cancer and reproductive abnormalities. It is thus a major challenge to limit the risks from waterborne microbial pathogens as well as byproducts of disinfectants in drinking water. Currently, the Guidelines for Drinking Water Quality set by the World Health Organization recommend a residual concentration of free chlorine of ≥ 0.5 mg/L [22]. As a general guideline for laboratory animals, chlorine concentration in drinking water should be maintained in the range of 0.5–10 mg/L, with 2.0–3.0 mg/L being standard [23, 24]. The chlorinated water (10.0 mg/L) administered to mice in this study did not significantly affect gut microbial communities, as determined by analysis of fecal bacteria. However, our data indicated that carcinogenesis in the colon was markedly altered by chlorinated water consumption, suggesting that the effect of chemicals or microorganisms on carcinogenesis in these *Apc* deficiency-induced spontaneous tumor models should be verified by standardizing chlorine concentrations in drinking water. Moreover, recent studies have suggested that human intestinal microbiota contributes to the onset of colorectal cancer not only by the pro-carcinogenic activities of certain pathogens but also by the release of specific metabolites [25, 26]. Our findings warrant further investigation in order to elucidate the biological significance and mechanisms underlying the link between chlorinated drinking water and carcinogenesis in the gut epithelium.

The *Apc* ^Min/+^ mouse is a well-established FAP model that has been used to test the potential carcinogenicity of dietary ingredients and chemotherapeutic agents before clinical trials. The *CPC;Apc* mouse carries an *Apc* allele in which *loxP* sites flank exon 14; these mice spontaneously develop colorectal tumors early in life at a high frequency [10]. In this model, tumors were observed in the gut epithelium from the distal ileum to the rectum in the region close to the *CDX2* promoter (*CDX2P9*.*5*). Similar to the high-volume tumors in the distal small intestines of the tap water group, we previously found that the vast majority of tumors in *villin-Cre;Apc* mice were located in the small intestine, although Cre-mediated recombination occurred in the epithelium throughout the small intestine and colon [10]. These results indicate that the presence of a large number of small intestinal adenomas may inhibit colorectal tumor growth, implying a negative association between small intestinal and colorectal tumors in mice.

While spectral karyotyping of tumors revealed aneuploidy, suggesting a role of CIN in tumor progression in the *CPC;Apc* mouse model [10], *CDX2P9*.*5-G19Cre;Apc* ^*flox/flox*^ and *CDX2P9*.*5-G22Cre;Apc* ^*flox/flox*^ mice provided evidence for MSI-induced colorectal carcinogenesis resulting from the elimination of both *Apc* alleles by Cre recombinase activity following a frameshift reversion mutation in a 19- or 22-guanine repeat sequence [11]. Our analysis of mono- and dinucleotide repeat markers demonstrated a hypermutable phenotype in tumors; *CDX2P9*.*5-G19Cre;Apc* ^*flox/flox*^ and *CDX2P9*.*5-G22Cre;Apc* ^*flox/flox*^ mice can therefore serve as models of MSI-derived colorectal cancer characterized by tumors with a hypermutable phenotype. MSI was originally discovered as a short product in an analysis of PCR amplicons from normal and tumor tissues [27]. Slippage during replication of a repetitive sequence creates a temporary insertion-deletion loop that is recognized and repaired by the DNA mismatch repair (MMR) system, which can result in frameshift mutations and downstream nonsense mutations that generate a truncated, non-functional protein. In order to recapitulate tumorigenesis with a hypermutable phenotype caused by MSI, knockout mouse strains have been generated for each of the Lynch syndrome genes (*MSH2*, *MLH1*, *PMS2*, and *MSH6*), although the principal type of tumor that develops in these mice is lymphomas and none of the heterozygous knockouts show a phenotype that is similar to that of Lynch syndrome patients [28–31].

Studies in pathogen-free mice have shown that enteric bacteria are required for suppressing colon cancer in some model systems [32, 33]. An analysis of the bacterial composition of feces showed that among the five species that were reduced in number in the chlorinated water group, *C*. *perfringens* has potential anti-tumorigenic activity. *C*. *perfringens* is a causal agent in food poisoning and spontaneous, non-traumatic gas gangrene, and is considered as an infectious agent; moreover, this species is present in higher numbers in the feces of human colorectal cancer patients as compared to healthy controls [34]. *C*. *perfringens* endotoxin was also shown to exert anti-tumor effects by binding to claudin-4 receptor-positive cancer cells [35]. Thus, the role of *C*. *perfringens* in colon carcinogenesis remains controversial; nonetheless, our results suggest that it may involve the modulation of LOH via CIN and not MSI.

Many studies have reported that infection with pathogenic bacteria can alter the host genome, producing double-strand breaks (DSBs) and other DNA modifications; even after DNA repair, chromosomes can remain scarred and cause genomic instability during the next round of cell division. *Escherichia coli* genotoxin induces DNA DSBs [36], while *Pseudomonas aeruginosa* induces single-strand breaks, DSBs, and oxidative DNA damage that activate a variety of DNA repair pathways [37]. The functional connection between the innate immunity and the DNA damage response has been demonstrated by many studies: for instance, *Enterococcus faecalis*—a Gram-positive intestinal commensal bacterium that produces extracellular superoxide—can polarize macrophages to induce a bystander effect that results in DSBs, tetraploidy, and CIN in target cells and can cause inflammation and colorectal carcinoma in interleukin (IL)-10 knockout mice [38, 39]. Since tumorigenesis in heterozygous *CPC;Apc* mutant mice depends on LOH of the remaining wild-type allele, this model can provide a useful benchmark for evaluating the role of potentially pathogenic bacteria in colorectal cancer.

Our study using two mouse models yielded observations that warrant a more detailed investigation. First, disinfectant byproducts such as trihalomethane and haloacetic acid are formed when chlorine reacts with naturally occurring organic matter present in water sources [40]. Some studies have found a minor association between exposure to chlorinated water containing these byproducts and the occurrence of bladder, rectal, and colon cancers, which was not addressed in this study. There is some uncertainty in terms of estimating the risk associated with chronic exposure to low doses of byproducts present in disinfected drinking water based on the results of high-dose toxicological studies. In this regard, our models may be useful for examining the effect of byproducts on tumorigenesis.

Second, the current study did not investigate whether the hypermutable phenotype of tumors in *CDX2P9*.*5-G19Cre;Apc* ^*flox/flox*^ and *CDX2P9*.*5-G22Cre;Apc* ^*flox/flox*^ mice arises from MMR deficiency (e.g., silencing of MMR-associated genes due to promoter mutation/deletion or hypermethylation), which is a characteristic of MSI tumors in humans. The study of MMR in a mouse model is limited by the fact that human genes associated with colorectal cancer contain coding microsatellites that are not present in mouse homologs. Furthermore, genes associated with colorectal cancer encode factors that are required for cell proliferation and survival—such as transforming growth factor β receptor 2, B cell lymphoma 2-associated X protein—and caspase-5, and it is therefore a challenge to generate mice with defects in these genes for cancer studies [41]. One way to address this problem is to generate mouse models by knocking down genes lacking coding microsatellites so as to recapitulate the histological phenotype of MSI (e.g., tumor-infiltrating lymphocytes, Crohn’s-like lymphocytic reaction, mucinous/signet-ring differentiation, and medullary growth pattern) [42]. A previous study found that the rate of frameshift mutations among 28 genes with long mononucleotide repeats in hypermutated tumors without *MLH1* silencing was nearly 15-fold higher than the rate of such mutations in non-hypermethylated tumors based on the Cancer Genome Atlas project plans [43]. Meanwhile, other studies have shown that > 90% of colorectal carcinomas have altered expression of genes involved in Wnt signaling [44, 45], providing further evidence of the importance of Wnt activation in the hypermutable phenotype. Therefore, despite the fact that *CDX2P9*.*5-G19Cre;Apc* ^*flox/flox*^ and *CDX2P9*.*5-G22Cre;Apc* ^*flox/flox*^ mice have no confirmed mutations/deficiencies in MMR-related genes, their hypermutable tumors in the proximal colon are associated with activated Wnt signaling, which recapitulates MSI tumors in humans.

Third, our data suggest a possible association between *C*. *perfringens* and tumorigenesis. The human intestine harbors around 100 trillion microorganisms, a number that is 10-fold higher than the total number of cells in the human body [46]. Identifying bacteria responsible for tumorigenesis is more challenging than detecting *H*. *pylori* in the stomach, an acidic environment in which few bacteria can survive. However, gut microbial communities are important for the maintenance of innate and adaptive immunity, while host-microbe symbiosis is necessary for gut homeostasis. Recent studies in *CPC;Apc* mice have revealed that IL-23, which is mainly produced by tumor-associated myeloid cells, promotes tumor growth and progression and induces the humoral IL-17 response. IL-17A exerts a pro-tumorigenic effect via its type A receptor [47] and activates extracellular signal-regulated kinase, p38 mitogen-associated protein kinase, and nuclear factor κB signaling in transformed enterocytes to promote early tumorigenesis [48]. However, the precise mechanisms by which microbiota contributes to immune system function remain to be elucidated.

In conclusion, we have shown here that chlorinated water influences the development of colorectal tumors with CIN, although further analysis is required to identify the underlying mechanism. Furthermore, assuming that tumorigenesis is directly or indirectly modulated by altered gut microbiota composition due to consumption of chlorinated water, our mouse models may also be useful for studying the interactions between these microorganisms, tumors, and immune responses.
